# Prevalence and socio-demographic factors associated with non-protective immunity against tetanus among high school adolescents girls in Nigeria

**DOI:** 10.1186/1824-7288-40-29

**Published:** 2014-03-17

**Authors:** Adebola E Orimadegun, Akinlolu A Adepoju, Olusegun O Akinyinka

**Affiliations:** 1 Institute of Child Health, College of Medicine, University of Ibadan, Ibadan, Nigeria; 2 Department of Paediatrics, College of Medicine, University of Ibadan, Ibadan, Nigeria

**Keywords:** Tetanus immunity, Schooling adolescents, Vaccination, Non-protective immunity

## Abstract

**Background:**

The low uptake of tetanus vaccine and its resultant high burden of tetanus in Nigeria suggest the need to improve routine and booster vaccination in children and adolescents. However, epidemiological evidence for vaccination in the adolescent age group needed for effective strategy and policy formulation is lacking. This study was carried out to determine the prevalence of protective immunity against tetanus and to identify risk factors for non-protective immunity among schooling adolescents.

**Methods:**

Using a three-stage sampling technique, 851 female adolescents were randomly selected from secondary schools in Ibadan, Nigeria. A pre-tested questionnaire was used to obtain data on demographic and socio-economic characteristics and history of tetanus vaccination. An immuno-chromatographic rapid test kit, “Tetanos Quick Stick” was used to test specific anti-tetanus antibody protective level in venous blood samples. Descriptive statistics, Chi-square and logistic regression analyses were done with level of significance set at p = 0.05.

**Results:**

Mean age of participants was 14.3 ± 1.9 years. Seroprevalence of protective immunity against tetanus was 38.1% and it significantly decreased with increasing age. More adolescents in public (65.4%) than private (44.7%) schools had non-protective level of immunity. A significantly increasing trend in the risk of non-protective immunity was observed with decreasing level of mothers’ education. Also, the Odds of non-protective level of immunity was significantly higher in public than private schools (OR = 2.14; 95% CI =1.39, 3.20) but lower among adolescents who had history of recent tetanus toxoid injection than those who did not (OR = 0.11 95% CI = 0.09, 0.22). However, no significant association was found between protective immunity against tetanus and parents’ marital status as well as family size.

**Conclusion:**

Protective immunity against tetanus among female adolescents was poor, more so in public schools and those who had not received vaccination a year prior to the study. Policy-makers need to consider the inclusion of immunization against tetanus in the school health programme.

## Background

Tetanus is one of the leading causes of death among hospitalised children in Nigeria especially in the neonatal age group. It accounts for about 13.6% of all child deaths and 26.4% of deaths in the neonatal age group in Ibadan, Nigeria [1]. Nigeria is one of the 16 countries in the African region with the highest numbers of neonatal deaths, accounting for 90% global neonatal tetanus cases [2]. Currently, only an estimated 47% of Nigerian infants receive the third dose of Diphtheria-Pertusis-Tetanus (DPT) vaccine and about 60% of women of child-bearing age have protective level of immunity against tetanus [3]. The slow pace of the uptake of health interventions by women of child-bearing age, 15 to 45 years, particularly vaccination against tetanus makes the elimination of tetanus unrealistic in the near future [4,5].

Neonatal tetanus is preventable. The World Health Organization (WHO) recommends that women of child-bearing age, should commence a 5-dose regimen of vaccination against tetanus as early as possible [6-8]. This comprises; first dose given at any time during the ages 15 to 45 years, a second dose four weeks later and a third dose given 6–12 months after the first two doses. Additional two doses, given at least one year apart further prolong the duration of immunity against tetanus. A previous study has shown that after the fifth dose, protective antibody levels may last for about 20 years [9] with resultant protection for the mother and her newborn who would be sufficiently protected against tetanus [8].

Though Nigeria started routine immunization against tetanus since 1979, there is no known programme aimed at ensuring that the recommended five doses of tetanus toxoid are given to adolescent girls before their first pregnancy. The current neonatal tetanus prevention programme principally focuses on giving only pregnant women two doses of tetanus toxoid injections during antenatal care (ANC) leaving out a large number who may not book at health facilities where vaccinations are provided [10]. The 2008 Nigeria Demographic Health Survey [11] showed that less than half of the eligible pregnant women get vaccinated. This is mainly due to the fact that a large number of pregnant women did not book for antenatal care where the vaccine was available and only about 35% of the women delivered in health facilities [11]. The female adolescents, especially those in schools, constitute a considerable number of the women in the child-bearing age and they could provide opportunity for delivering tetanus vaccination to prospective mothers. However, Nigeria has no formal or structured health system that caters for the adolescent population in place and the current immunization policy [10] does not sufficiently address vaccination for the adolescents. Thus, a significant proportion of eligible women of child-bearing ages neither receive vaccination against tetanus before nor during pregnancy thereby putting them and their newborns at risk of tetanus. The only study that has assessed tetanus immunity among Nigerian adolescents was conducted almost about 20 years ago [12]. Brabin and Colleagues determined tetanus antibody seropositivity in adolescent girls from Nigeria as part of a multinational study and 54.7% of adolescent girls were reported as seronegative [12]. In that study, data from Nigeria were obtained among adolescents living in rural communities of Port Harcourt, South-eastern Nigeria. The authors proposed that booster vaccination in early adolescence may protect women in their reproductive ages [12].

Two recent studies demonstrated some socio-demographic factors associated with uptake of vaccines in infancy and early childhood but not adolescents [4,5]. Brown and Oluwatosin [4] showed that maternal education (secondary school or more), being married and living in urban areas of Nigeria were associated with improved immunization among children aged 12–24 months. Antia [5] also reported that “the woman being the sole provider for her family was associated with a higher likelihood of fully immunizing the child” in Nigeria. The question of, what socio-demographic factors distinguish female adolescents with non-protective level of immunity from those without, remains unanswered in published literature. In view of this, the present study was conducted to evaluate immunity against tetanus among adolescent girls with a view to providing evidence for or against the practicability of tetanus immunization in this age group in Nigeria.

## Methods

### Study design and setting

This study was cross-sectional in design. It involved adolescent girls selected from secondary schools, who were residents of Ibadan North Local Government Area (IBNLGA), Nigeria. The IBNLGA has a population of 306,795 [13], mainly Yoruba-speaking people and has urban and slum communities. These communities are divided into twelve geo-political wards, defined on the basis of the populations and contiguity of communities. This study was conducted between June and August, 2012. There were 28 public and 32 private registered secondary schools in IBNLGA as at the time of this study.

### Study population and sampling method

This study was targeted at female students aged 10 to 19 years. Students were eligible to participate in the study if they had resided in IBNLGA for more than a year. A three-stage random sampling technique was used to select geo-political wards, schools and students. The first stage involved the division of IBNLGA into four quadrants based on the geographic map and two wards were randomly selected from the list of wards in each quadrant. In stage two, all the private and public schools were listed with one private and one public school randomly selected from the list resulting in a total of eight schools. In stage three, a sampling frame of the adolescent girls in the selected schools was compiled and participants were randomly selected from the list. The number of participants from each school was based on proportion as follows:

$$\begin{array}{l} \mathrm{Participants}\;\mathrm{in}\;\mathrm{each}\;\mathrm{school}\\ \;=\frac{\left(\mathrm{Number}\;\mathrm{of}\;\mathrm{adolescent}\;\mathrm{girls}\;\mathrm{in}\;\mathrm{each}\;\mathrm{school}\times \mathrm{Sample}\;\mathrm{size}\right)}{\mathrm{Total}\;\mathrm{number}\;\mathrm{of}\;\mathrm{adolescent}\;\mathrm{girls}\;\mathrm{in}\;\mathrm{all}\;\mathrm{the}\;\mathrm{selected}\;\mathrm{schools}} \end{array}$$

### Sample size justification

It was postulated that the prevalence of protective immunity against tetanus among school girls was 45.3% based on the report by Brabin and colleagues [12]. Using the formula for estimating sample size for cross sectional study, 381 was calculated as the minimum number of participants required at 95% level of confidence. A design effect factor of 2 was assumed, the estimated sample size was multiplied by 2 giving a total of 762 participants as minimum sample size [14]. However, post-hoc power calculation revealed that using an effect size of 5%, enrolling 851 adolescent girls gave a study power of over 80% at the same level of confidence.

### Instruments and data collection procedure

A questionnaire developed for the purpose of this study was pre-tested in two secondary schools located outside IBNLGA. Thereafter the instrument was revised to ensure construct validity. Tetanos Quick Stick (TQS) (Gamma, Angleur, Belgium), a validated immunochromatographic test [15-17] was carried out on each participants by one of the investigators. The questionnaire contained information on socio-demographics, tetanus immunization history and previous tetanus infection in ≤1 year. Those who tested negative to TQS were referred to the nearest primary health centre within IBNLGA for tetanus toxoid vaccination.

### Data analysis

Data obtained were analysed using SPSS for Window 17.0 (SPSS, Inc., Chicago, IL USA). Participants’ socio-economic class scores were determined based on the occupations and educational attainments of the parents using the classification method earlier reported by Oyedeji [18]. This method determines the mean of scores from education and occupation of both parents and rounded up to the nearest whole number. The number thus obtained was the social class assigned to the child. In this study, score of 1 point was assigned upper class; 2 and 3 points were grouped as middle class while 4 and 5 points were regarded as lower class. Prevalence of non-protective immunity was calculated for the different participants’ characteristics. Bivariate analysis was done using the Chi-square test for associations. Logistic regression was used to determine independent predictors of non-protective immunity against tetanus among factors that were found to be significant at bivariate analyses level. The possible interaction between age and school types was also assessed by including the interaction term in the model. The odds of non-protective immunity for various variables were assessed using Odd Ratios (OR) and 95% Confidence Intervals (CI) at p = 0.05.

### Ethical consideration

Approval to carry out the study was obtained from the Oyo State Ministry of Health Research Ethical Review Committee (reference number: AD 13/479/215). Written informed consent was obtained from each of the participant’s parents or guardians after the purpose of the study was explained to each of them in the language they understood, mainly Yoruba. Also, a written permission to conduct the study was also obtained from the State Commissioner of Education while the Principals of the selected schools were met to obtain their verbal permission as well. Serial numbers and codes were used to identify participants and schools. The participants were assured that their responses would not be reported individually but as part of an overall study report or scientific publications.

## Results

### Demographic characteristics of participants

There were 851 participants; 710 (83.4%) and 141 (16.6%) adolescents from public and private schools, respectively. Most of the participants (90.1% in private and 90.4% in public schools), belong to the Yoruba ethnic group while others were non-Yoruba (51 Ibos, 25 Hausas, 4 Urhobo and 2 Efik). Majority (86.0%) of the participants were from homes where parents were living together, while few (2.0%) had single parents. Those from families with more than four children were 88.5%. Others were from families with four or fewer children. Almost two-thirds (62.4%) of participants were in the range of 2nd to 4th born of their mothers, while 28.8% were 1st born and the others were beyond the 4th position (8.8%) among mother’s children.

**Figure 1:**
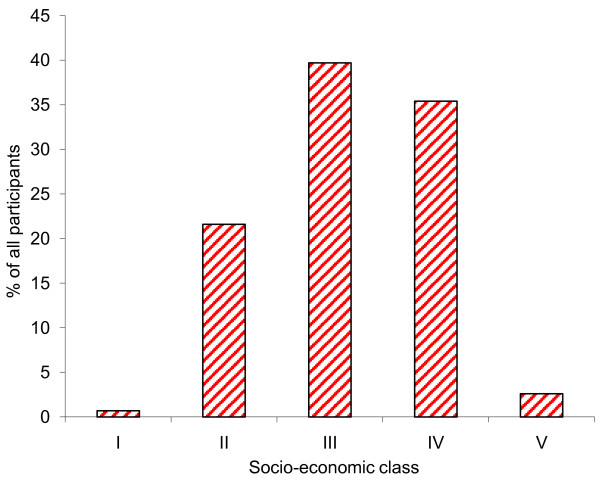
Distribution of adolescents by parents’ socio-economic class.

Mean age of adolescents recruited from public schools (14.4 ± 1.9 years) was significantly higher than those from private schools (13.6 ± 1.6 years; p <0.001). Other demographic characteristics of the participants were as shown in Table 1. About three quarters (74.1%) of the adolescents had domestic animals such as goats, sheep, cow and other livestock being reared near their houses while 52.3% of the participants’ family or neighbours owned livestock. The distribution of participants according to their parents’ socio-economic class (Figure 1) revealed that 39.7% belonged to the socio-economic class III and the least represented socio-economic class was I (0.7%).

**Table 1:** Demographic characteristics of adolescents in private and public schools in Ibadan, Nigeria

**Characteristics**	**All participants (851)**		**Public Sschools (710)**		**Private schools (141)**		P*
	**n**	**%**	**n**	**%**	**N**	**%**	
**Ethnic groups**							
Yoruba	769	90.4	642	90.4	127	90.1	0.897
Non-Yoruba	82	9.6	68	9.6	14	9.9	
**Parents marital status**							
Single parent	17	2.0	14	2.0	3	2.1	<0.001
Married living with spouse	732	86.0	597	84.1	135	95.7	
Divorced/separated	102	12.0	99	13.9	3	2.1	
**Number of mother’s children**							
1 – 4	98	11.5	70	9.9	28	19.9	0.001
>4	753	88.5	640	90.1	113	80.1	
**Position among mother’s children**							
First	245	28.8	198	27.9	47	33.3	0.418
2^nd^ to 4^th^	531	62.4	448	63.1	83	58.9	
Beyond 4^th^	75	8.8	64	9.0	11	7.8	

*p values for comparison between private and public schools.

### Tetanus immunization history

Only 39 (4.6%) of the participants received tetanus immunization in the one year prior to the study; of this, 9 (23.1%) were in private schools and 30 (76.9%) in public schools. Majority (65.4%) of the 39 mentioned “wound/injury” as the reason for being given tetanus toxoid injection. Participants who had received tetanus toxoid injections: 16 (41.1%), 10 (25.6%), 8 (20.5%) and 5 (12.8%) were given at primary health centres, private clinics or hospitals, mission hospitals and patent medicine stores respectively. However, only 282 (33.1%) out of the 851 could produce their immunization cards.

### Prevalence and risk of non-protective immunity against tetanus

**Figure 2:**
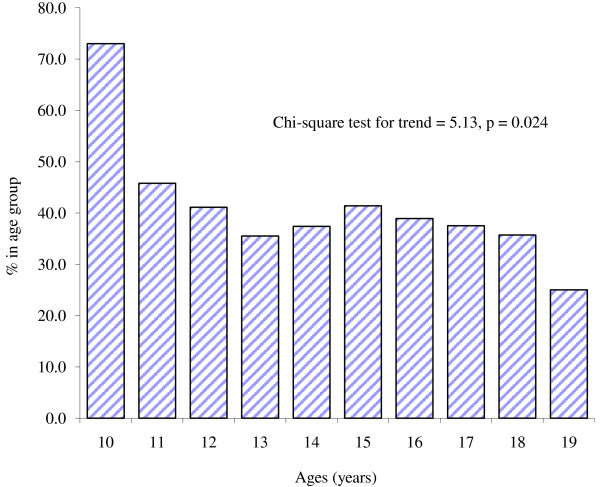
Trends of age-specific seroprevalence of protective immunity against tetanus.

Of the 851 adolescents, 324 tested positive to “Tetanos Quick Stick” test giving a seroprevalence of protective immunity against tetanus of 38.1%. Seroprevalence of protective immunity against tetanus among adolescents attending private school of 55.3% (78/141) was significantly higher than 34.6% (246/710) of those in public schools (unadjusted OR = 2.34; 95% CI = 1.62, 3.37; p < 0.001). Figure 2 shows the trend of age-specific seroprevalence of protective immunity against tetanus. There was a significant trend as the prevalence of protective immunity against tetanus decreased rapidly with increasing age till age 13 years (35.0%), (p = 0.024).

Table 2 shows the prevalence of protective immunity against tetanus in relation to mothers’ educational status. The participants whose mothers obtained university degrees (46.9%) had the highest prevalence of protective immunity against tetanus. Maternal education was significantly associated with protection against immunity (p = 0.013). However, comparing the prevalence of protective immunity against tetanus among other categories of educational attainment with the University degree category, the risk of non-protective immunity against tetanus was significantly higher among those whose mothers had post-secondary certificate but not university (unadjusted OR = 1.69; 95% CI = 1.17, 2.43) and Secondary School or Grade II certificate (unadjusted OR = 1.79; 95% CI = 1.25, 2.56). A significantly increasing trend in the risk of non-protective immunity was also observed with decreasing educational attainment (p = 0.014).

**Table 2:** Prevalence of protective immunity against tetanus by mothers’ education

**Mother level of education**	**Total**	**Level tetanus immunity**				**OR (95% CI)**	**P**
		**Non-protective**		**Protective**			
		**N**	**%**	**n**	**%**		
No formal education	18	11	61.1	7	38.9	1.39 (0.52, 3.69)	0.680
Modern 3 and Primary 6 Certificate	90	57	63.3	33	36.7	1.52 (0.93, 2.50)	0.120
Secondary School or Grade II certificate	251	168	66.9	83	33.1	1.79 (1.25, 2.56)	0.002
Post-Secondary certificate, not University	236	155	65.7	81	34.3	1.69 (1.17, 2.43)	0.006
University graduates*	256	136	53.1	120	46.9	1	-

Chi Square test for trend = 6.062; p = 0.014.

*Reference category, OR = Odds Ratio (for being negative), CI = Confidence Interval.

Seroprevalence of tetanus immunity by parents’ socioeconomic class as well as risk of being seronegative to TQS were as shown in Table 3. There was no significant association between socio-economic class and level of tetanus immunity. The odds of non-protective level of immunity among those in the high socio-economic class were not significantly different from middle and lower classes respectively.

**Table 3:** Seroprevalence of tetanus immunity by socioeconomic class

**Socioeconomic class**	**Level of tetanus immunity**				**Total**	**P**	**OR (95% CI)**
	**Non-protective**		**Protective**				
	**n**	**%**	**N**	**%**			
High*	123	64.7	67	35.3	190	-	1
Middle	206	60.9	132	39.1	338	0.441	0.85 (0.59, 1.23)
Low	198	61.3	125	38.7	323	0.495	0.86 (0.59, 1.25)

Chi Square test for trend = 0.47; p = 0.495.

*Reference category, OR = Odds Ratio (for being seronegative), CI = Confidence Interval.

Table 4 shows the prevalence of tetanus immunity by types of school, parents’ marital status, family size, position among mother’s children and tetanus immunization history. There was a significant association between age and protective immunity against tetanus. The regression model showed that a year increase in age increases the odds of non-protective immunity by 9% (adjusted OR = 1.09; 95% CI = 1.02, 3.07). Adolescents in public schools had significantly higher odds of having non-protective level of immunity against tetanus compared with those in private schools (adjusted OR = 2.14; 95% CI = 1.39, 3.20). Using “married parents living together with their spouses” as the reference category, there was no significant differences in odds of non-protective immunity against tetanus among participants with single parents (adjusted OR = 1.28; 95% CI = 0.31, 4.12) and divorced/separated parents (adjusted OR = 1.13; 95% CI = 0.49, 2.47) compared with children whose parents were together.

**Table 4:** Prevalence of tetanus immunity by parents’ marital status, Ethnicity, family size, and tetanus immunization history

	**N**	**Level tetanus immunity**				**UOR (95% CI)**	**AOR (95% CI)**
		**Non-protective**		**Protective**			
		**n**	**%**	**n**	**%**		
Age (years)	851	527	61.9	324	38.1	1.21 (1.09, 2.52)	1.09 (1.02, 3.07)
Type of school							
• Public schools	710	464	65.4	246	34.6	2.34 (1.62, 3.37)	2.14 (1.39, 3.20)
• Private schools	141	63	44.7	78	55.3	1	1
Parents marital status							
• Single parents	17	12	70.6	5	29.4	1.54 (0.54, 4.41)	1.28 (0.31, 4.12)
• Married living with spouse	732	446	60.9	286	39.1	1	1
• Divorced/separated	102	69	67.6	33	32.4	1.34 (0.86, 2.08)	1.13 (0.49, 2.47)
No. of mother’s children							
• 1 – 4	98	60	61.2	38	38.8	0.97 (0.63, 1.49)	0.55 (0.34, 1.91)
• >4	753	467	62.0	286	38.0	1	1
Child’s position^a^							
• First	245	146	59.6	99	40.4	1	1
• 2^nd^ – 4^th^	531	332	62.5	199	37.5	1.13 (0.83, 1.54)	1.09 (0.84, 1.53)
• Beyond 4^th^	75	49	65.3	26	34.7	1.28 (0.74, 2.19)	1.16 (0.57, 2.09)
History of recent TT injection							
• Yes	39	7	17.9	32	82.1	0.12 (0.05, 0.28)	0.11 (0.09, 0.22)
• No	812	519	63.9	293	36.1	1	1
Age and Type of school^b^						-	1.01 (0.69, 1.18)

^a^Among mother’s children, ^b^interaction term, UOR – Unadjusted Odds ratio, AOR – Adjusted Odds ratio.

Also, regarding the number of mother’s children, the odds for non-protective immunity among participants whose number of mother’s children were 1 to 4 than those > 4 (adjusted OR = 0.55; 95% CI = 0.34, 1.91) was not significantly different from others (p = 0.966). Moreover, the odds of non-protection for tetanus immunity for those who were in the 2^nd^ – 4^th^ positions (adjusted OR = 1.09; 95% CI = 0.84, 1.53) and beyond the 4^th^ position (adjusted OR = 1.16; 95% CI = 0.57, 2.09) were not significantly different from those who are first. About eighteen percent (17.9%) of the participants who did received recent tetanus toxoid injection had non-protective immunity compared to 63.9% of those who had no history of recent tetanus toxoid injection (p < 0.001). The risk of non-protective immunity in the group with recent history of tetanus toxoid injection was significantly lower than those who gave no history of recent tetanus injection (adjusted OR = 0.11; 95% CI = 0.09, 0.22). Though an association was found between age and school types, there is no interaction between the effects of these two explanatory variables when a model with an interaction term between age (in years) and school types (private or public) was fitted.

## Discussion

This study revealed that the prevalence of protective immunity against tetanus among adolescent girls was 38.1%, about two-third of the participants had non-protective level of immunity, more adolescent in private than public schools were protected against tetanus, prevalence of protection against tetanus decreased with increasing age and mothers who have at least tertiary education were more likely to have female adolescents with protective immunity against tetanus than others. The obvious implication of the low prevalence of protective immunity against tetanus is that most of the participants probably received neither tetanus toxoid injection nor any effective tetanus vaccination many years after infancy. Only 3.1% claimed they had tetanus injections for various reasons within one year prior to the study.

The prevalence obtained from the present data is lower than 45.3% earlier reported in a multinational study involving girls selected from a rural community of Port Harcourt, Nigeria [12]. However, apart from the fact that those girls who participated in the study by Brabin and Colleagues [12] were selected from rural community, Enzyme-linked immunosorbent assay (ELISA) method was used to determine tetanus antibody level against TQS used the present study. Also the prevalence of protective immunity against tetanus in the present study is far lower than 88.9% reported by Dinelli et al., [19] among female adolescents in Brazil. Again, ELISA method was used in that study. Unlike the female adolescents who participated in the current study, all the Brazilian female adolescents were recently immunised against tetanus. A study in Turkey had attributed a high prevalence of protective immunity against tetanus to the success of Expanded Programme on Immunization, which recommends two booster doses of tetanus vaccine after infancy including a second dose during adolescence [20]. It is worth noting that only about a third of the participants in our study, produced immunization cards and only a small proportion of the adolescents had received immunization within a year prior to the study. Therefore, it is difficult to make any valid comparisons between the data from Turkey [20] and Ibadan, Nigeria. It is also difficult to compare findings from the present study with others in the literature because many of these studies investigated prevalence of tetanus immunity in adults and none was carried out in the adolescent population [21-23].

Another important finding from the present study was that the prevalence of protective immunity against tetanus decreased with increasing age among the adolescent population. Naturally, immunity against tetanus declines with time. Individuals require multiple doses to attain protection against tetanus and three doses of tetanus toxoid given to infants as part of DPT only confer protection till about the age of 5 years, it is not unexpected that by adolescence very few will have detectable level of immunity against tetanus [24]. The highest level of immunity was observed in the younger segments of the population, who may have been covered by routine immunization. The implication of extremely low tetanus immunity towards the end of adolescence is that most women will enter child-bearing age with little or no protection. This may explain the high burden of maternal and neonatal tetanus currently being experienced in Nigeria.

The difference in the seroprevalence of protective immunity against tetanus between private and public schools was not surprising, most private schools are largely populated by children from high socioeconomic class of the society and their vaccine uptakes are likely to be better than those from lower socioeconomic status [25,26]. However, when the female adolescents who participated in the study were stratified by their parents’ socio-economic class there was no significant association between seroprevalence of protective immunity against tetanus and socio-economic class. This finding is in keeping with the findings by Inandi and colleagues [27] which showed that there was no association between immunity against tetanus and socioeconomic status among children in Turkey. Moreover, just as in the report by Inandi and colleagues [27], the present study also demonstrated an increased risk of low immunity against tetanus in female adolescents whose parents were less educated. It was also observed from this study that there was no association between protective immunity against tetanus and recent history of tetanus toxoid injection. Only a few of the participants gave history of recent tetanus toxoid injection. Immediate and long-term responses to tetanus toxoid injection depend on individual’s previous immunity. Regarding the association between maternal level of education and prevalence of protective immunity among participants, there is no conceivable biological explanation for the lowest protective immunity found among adolescents in the groups of mothers with secondary or post-secondary certificates.

This study has provided data that may influence child health and clinical practice. Among other reasons, the persistence of tetanus as the cause of high proportion of deaths among Nigerian children in spite of availability of vaccines and past efforts made towards immunization against tetanus and the high fatality rate (over 50%) of tetanus underscores the need to assess the effectiveness and extent of protection among adolescent population. Universal immunization of school adolescents is one of the promising strategies that may reduce the burden of tetanus in countries. However, the current Nigerian healthcare system appears not suitably structure to deliver such service. Though this approach is currently been employed in the fight against vitamin A deficiency as well as polio and it appears to be yielding promising results [11]. To apply the same approach in the reduction of tetanus burden in Nigeria, there is the need to further assess its acceptability among school children and their caregivers including parents and teachers.

Two important factors limit the generalisability and interpretations of findings from this research. First, the fact that those female adolescents who participated in this study were recruited from the metropolitan area of Ibadan and from schools may limit the generalisation of the findings to those in rural area and other parts of Nigeria. One can only speculate that the poor protective level of immunity may be worse in the rural and hard-to-reach communities in Nigeria. Second, the TQS gives semi-quantitative assessment of immunity level, thereby restricting further stratification of protection level or lack of it in the study population.

## Conclusion

This study has shown that the prevalence of non-protective level of immunity against tetanus among Nigeria adolescent girls is high. Age and types of school are important factors to be considered in tackling the menace of low tetanus protection among women of reproductive age. Specific plans of action need to be developed to reach the adolescents population of urban areas. Given the multifaceted challenges inherent in health programmes, an inter-sectorial approach is necessary. For instance, efforts should be made in the existing health facilities to use any contact with eligible adolescents as an opportunity to immunise against tetanus. The need to commence school-based immunization campaign against tetanus for female adolescents in Nigeria is also suggested.

## Competing interests

The authors declare that they have no competing interests.

## Authors’ contributions

AEO conceptualised and designed the study, analysed the data and participated in writing the manuscript. AAA participated in the design of the study, supervised collection of data and contributed to writing the manuscript. OOA contributed to design of the study, interpretation of results and writing of the manuscript. All authors read and approved the final manuscript.
